# A Clinical Retrospective Study of Distal Extension Removable Partial Denture with Implant Surveyed Bridge or Stud Type Attachment

**DOI:** 10.1155/2017/7140870

**Published:** 2017-04-09

**Authors:** Eun-Bin Bae, Seong-Jong Kim, Jae-Won Choi, Young-Chan Jeon, Chang-Mo Jeong, Mi-Jung Yun, So-Hyoun Lee, Jung-Bo Huh

**Affiliations:** Department of Prosthodontics, Dental Research Institute, Institute of Translational Dental Sciences, BK21 PLUS Project, School of Dentistry, Pusan National University, 49 Pusan University-Ro, Yangsan-Si, Gyeongsangnam-do 50612, Republic of Korea

## Abstract

This study was performed to make comparative analysis of the clinical findings between the two different types of the implant-assisted removable partial dentures: removable partial dentures using implant surveyed bridge as an abutment (ISBRPD) and overdenture type of removable partial denture using implant attachment (IARPD). Implant cumulative survival rate, marginal bone resorption, probing depth, peri-implant inflammation, bleeding, plaque, calculus, and complications were evaluated on 24 patients who were treated with implants in conjunction with removable partial denture and have used them for at least 1 year (ISCRPD: *n* = 12; IARPD: *n* = 12). There was no failed implant and all implants were functioning without clinical mobility. Marginal bone loss of ISCRPD (1.44 ± 0.57 mm) was significantly lower than that of IARPD (*p* < 0.05). There was no significant difference in probing depth, peri-implant inflammation, bleeding, and plaque between the two groups (*p* > 0.05), while the calculus was significantly more observed in ISCRPD group than in IARPD group (*p* < 0.05). The retention loss of IARPD was the most common complication. Within the limits of the present study, it was found that well-planned ISBRPD was clinically appropriate. Longitudinal and systematic clinical studies are necessary to confirm these results.

## 1. Introduction

Treatment with removable partial denture is an effective treatment for partially edentulous patients who are unable to obtain sufficient retention and stability for functional and esthetic restoration [1]. If a partially edentulous patient uses a distal extension removable partial denture for a long term, complications may occur such as alteration of the occlusal plane or loss of vertical dimension due to bone resorption under the supportive tissue and artificial teeth wear [2, 3]. Also, changes in the occlusal plane and the occlusal aspect may cause an excessive lateral force on the residual abutment, which may also have an adverse effect on prognosis of the abutment [2, 4]. There are several cases that reported that the partial denture using a small number of implants supporting the posterior edentulous area could overcome this phenomenon by protecting the remaining soft tissue and hard tissue and by improving support, retention, and stability to manufacture functionally satisfying implant-assisted removable partial denture [4–6]. Such implant-assisted removable partial dentures have been categorized into implant-supported and implant-retained removable partial dentures according to connection method. However, the term of “implant-assisted removable partial denture” is more widely used since Schneid and Mattie [7] introduced it as a more comprehensive concept.

General implant-assisted removable partial denture, a type of implant overdenture, has the advantage of prolonging the prognosis of the residual abutment and reducing the costs compared to fixed implant prostheses. In addition, compared to the general removable partial denture, patient satisfaction as well as esthetic aspects was improved by omitting the clasp [8–11].

Also, Ohkubo et al. [9] reported that the efficiency of the masticatory function was highly increased when support is given to the distal extension area using implants. Del'Arco Pignatta Cunha et al. [12] reported that when comparing the force applied to the abutment while the location of implant moved from the last molar area to the premolar area, the force distribution was more favorable as the implant was placed closer to the incisor area. Furthermore, the case reports and long-term clinical studies that placed implants in front of the edentulous area showed satisfying results [13–15]. Among the attachments used in the implant-assisted removable partial denture, Locator® is one of the most widely used because of its small limited vertical space and easy replacement of accessories [11, 15].

Recent proposals introduce that the implant-assisted removable partial denture has been applied strategically to a place with favorable bone support in partially edentulous or fully edentulous patients and use implant-supported fixed prosthesis as a partial denture abutment to fabricate the traditional partial denture [16–19]. The use of the implant surveyed bridge as an abutment for partial denture increases the stability of the denture through a small number of implants while having the function and esthetic merit of the fixed prosthesis, which could enhance the clinical outcome and patient satisfaction. However, a few studies have investigated the use of implant surveyed bridge as an abutment for removable partial denture. In addition, such studies were only limited to case reports. Therefore, further clinical studies are required.

This study was performed in the Department of Prosthodontics, Pusan National University Dental Hospital, to evaluate the efficacy of implant-assisted partial denture using surveyed bridge through clinical assessment of implant survival rate and prosthesis complications in two different types of partial denture cases. One type is the surveyed bridge fabricated by implants placed on partially edentulous sites, used as an abutment and restored with a distal extension partial denture, and the other case is the overdenture type partial denture made by connecting Locator implant attachment on top of the implants.

## 2. Materials and Methods

### 2.1. Research Subjects

Among the patients treated with the implant-assisted removable partial denture manufactured in Department of Prosthodontics, Pusan National University Dental Hospital, from 2008 to 2016 for 8 years, the patients who used the denture for at least 1 year under functional loading with regular checkup were subjected in this study. The subjects of this study were selected from the patients who were deemed to need implants for additional support and stability in Kennedy class I or class II type distal extension removable partial denture, where the antagonists are mainly complete or partial denture. The cases with more than 10° in the direction of denture insertion and the implant placement were excluded among the patients with 2 or more implants placed to construct a surveyed bridge or attachment being connected and Locator implant attachment being used. In addition, patients with systemic diseases, such as uncontrolled diabetes, anticancer therapy, bleeding disorder, immune diseases, hormones, which could affect the treatment of partial dentures, and patients with alcohol or drug addiction and patients who did not undergo the posttreatment regular checkup were excluded from the analysis. A total of 24 patients (male: 6; female: 18) who met these criteria underwent this study under the review of the Bioethics Committee of Pusan National University (IRB number: PNUDH-2015-018) (Table 1).

### 2.2. Classification of Partial Denture

**Figure 1:**
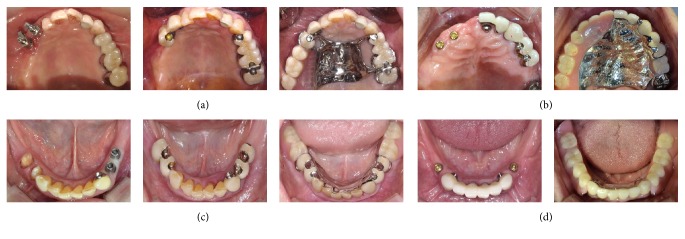
Intraoral view of the study groups. (a, c) ISBRPD group, removable partial denture with fixed implant surveyed bridge; (b, d) IARPD group, removable partial denture with Locator implant attachment.

A total of 24 patients were divided into two groups according to the clinical application method of implant-assisted removable partial dentures. The partial dentures are classified as the Implant Surveyed Bridge Removable Partial Denture (ISBRPD) group which was made in conventional design after a fixed surveyed prosthesis for partial denture abutment was made on top of the implant and the Implant Attachment Removable Partial Denture (IARPD) group (*n* = 12) which was made as overdenture type using the implant and Locator implant attachment (Zest Anchors Inc., Escondido, CA, USA) (Figure 1).

### 2.3. Clinical Examination

The following criteria were evaluated with reference to clinical examination and radiographs from the date of delivery of the implant-assisted partial denture to the final visit date.

#### 2.3.1. Implant Survival Rate

Implant survival rate was evaluated according to the criteria presented by Cochran et al. [20]. The evaluation criteria were the following: (1) no persistent discomfort such as pain, foreign body sensation, and abnormal sensation, (2) no persistent symptoms of peri-implant infection, such as pus discharge, and no relapse of such symptoms, (3) no clinical mobility of the implants, and (4) no radiographic lucency around the implant and no rapidly progressing bone loss.

#### 2.3.2. Implant Marginal Bone Resorption

Radiographs were taken using the paralleling technique with a portable radiographic device (PORT II, Genoray Co., Seongnam, Korea). The implant length and marginal bone level (distance from the implant platform to the top of the marginal bone) were measured using i-Solution (Olympus B × 51; Olympus Inc., Tokyo, Japan) and then the amount of marginal bone resorption was calculated by comparing the implant length [21].

#### 2.3.3. Probing Depth

The probing depth was measured at four points (mesial, distal, buccal, and lingual) around the implant in parallel with the long axis of the implant with Merritt-B periodontal probe, and then mean value was calculated [22].

#### 2.3.4. Peri-Implant Inflammation

Using the Löe-Silness index [23], scores from 0 to 3 were assigned according to the inflammatory state around the implant.

#### 2.3.5. Bleeding Index

The bleeding tendency was assessed using a Merritt-B periodontal probe according to the criteria proposed by Mombelli et al. [24].

#### 2.3.6. Plaque Index

According to the criteria of Mombelli et al. [24], the plaque attached to the surface of the implant was measured and a score from 0 to 3 was assigned.

#### 2.3.7. Calculus

Depending on the presence or absence of calculus, a score of 0 or 1 was given.

#### 2.3.8. Complication

The total treatment frequency after prosthesis delivery was classified as (1) denture related, such as resin base fracture, artificial tooth fracture, frame fracture, and new prosthesis production, (2) implant related such as screw loosening and replacement of locator, and (3) soft tissue related such as sore spot and soft tissue proliferation.

### 2.4. Statistical Analysis

The independent* t*-test was performed on implant marginal bone resorption and probing depth. The significance of peri-implant inflammation, bleeding index, plaque index, calculus, and complications was confirmed by chi-square test. Pearson's chi-square test was used to correlate the amount of marginal bone resorption, probing depth, and plaque index. All statistics were based on SPSS ver. 21.0 (SPSS Inc., Chicago, IL, USA) at a significance level of 5%.

## 3. Results

### 3.1. Implant Survival Rate

There were a total of 24 patients with implant-assisted partial denture; 53 implants were placed: 25 implants in the ISBRPD group and 28 implants in the IARPD group. Of these, 22 implants (ISBRPD group:* n* = 10; IARPD group:* n* = 12) were under functional loads from 12 to 24 months after placement of the partial dentures, and 14 implants were under occlusal load for 25 to 36 months (ISBRPD group:* n* = 8; IARPD group:* n* = 6). There were 17 implants under occlusal load for more than 36 months (ISBRPD:* n* = 7; IARPD:* n* = 10). The mean duration of loading was 26.7 months in the ISBRPD group and 23.5 months in the IARPD group. There were no double failed implants and all implants were normal in function without clinical mobility (Table 2).

### 3.2. Implant Marginal Bone Resorption and Probing Depth

The mean values and standard deviations of implant marginal bone resorption and probing depth are shown in Table 3. The ISBRPD group showed 1.44 ± 0.57 mm, a significantly lower implant marginal bone resorption than the IARPD group (*p* < 0.05), and there was no significant difference in probing depth between the two groups (Table 3).

### 3.3. Peri-Implant Inflammation and Bleeding Index

In both groups, normal condition was the most dominantly observed state and mild inflammation was the next dominant condition observed. Moderate and severe inflammation was not observed. Mild inflammation was not significantly different between the IARPD group (21.4%) and the ISBRPD group (21.7%) (*p* > 0.05). For the bleeding index, there was no bleeding in the ISBRPD group and the petechia was the most frequent type of bleeding occurring in the IARPD group. The frequency of petechia type of bleeding was slightly higher in the IARPD group (39.3%) than in the ISBRPD group (26.1%), but the difference was not significant (*p* > 0.05) (Table 4).

### 3.4. Plaque Index and Calculus

No plaque index was observed in the ISBRPD group and the score of 1 was the most frequently observed while probing in the IARPD group, but the difference in the plaque index was not significant. The calculus in the ISBRPD group (30.4%) was significantly higher (*p* < 0.05) than that in the IARPD group (3.6%) (Table 4).

### 3.5. Complications

Complications occurred more frequently in the IARPD group than in the ISBRPD group. Locator male replacement (64%) and denture repair (22%) were frequent in the IARPD group, and denture relining (67%) and denture repair (33%) were frequent complications in the ISBRPD group (Table 5). Complications were mainly necessity of maintenance due to the replacement of the Locator male in the IARPD group.

## 4. Discussion

Long-term clinical studies of removable partial dentures suggest that the stable design and periodic checkup are important factors affecting the outcome [25–27]. The use of implant-assisted removable partial denture using an implant surveyed bridge or attachment in distal extension removable partial denture reduces the width of the edentulous area and allows the practitioners to design more stable partial denture.

All 53 implants of 24 patients selected for this study were the external connecting type and 2 or more of these implants were implanted in front of the partial edentulous area with favorable bone support. The ISBRPD group used surveyed bridge supported on implants and the IARPD group used Locator implant attachment on top of each implant. According to the criteria of Cochran et al. [20], all implants had a high survival rate without any mobility and discomfort during the observation period.

The IARPD group showed higher values in implant marginal bone resorption than in the ISBRPD group (*p* < 0.05). Adell [28] reported that the marginal bone loss in a successful implant disappears after 1 year of abutment connection, so the prognosis should be assessed after 1 year. In the study by Adell et al. [29], the marginal loss was between 1 and 1.5 mm, with an average of 1.2 mm during the first year of abutment connection, and 0.1 mm of marginal bone loss per year was observed thereafter. The average follow-up periods of placed implants were similar in the two groups: 26.7 months in the ISBRPD group and 23.5 months in the IARPD group. It is assumed that the amount of marginal bone resorption is less in the ISBRPD group, because the implants are connected to and fixed by surveyed bridge, which reduces stress, causing microdamage by efficiently dispersing the load generated during mastication [30].

The two groups showed no significant difference in the plaque index, but the ISBRPD group (*p* < 0.05) had higher calculus index. This result could have been expected because it is difficult to maintain oral hygiene due to the characteristics of fixed prosthesis and it is relatively easier to manage oral hygiene in the IARPD group which is a solitary type [31]. Implant surveyed bridge requires thorough care of oral hygiene especially on the proximal sides and it is easier to acquire plaque and calculus in the margins of the prosthesis because it is cemented to the customized abutment.

There was no significant difference between the two groups in terms of peri-implant inflammation and bleeding index, but the IARPD group (*p* > 0.05) had higher bleeding index. McKinney Jr. et al. [32] reported that radiographic marginal bone loss and an increase in the probing depth were related to bleeding index through a scanning electron microscope. However, Villata and Baelum [33] pointed out that the reproducibility and accuracy of the probing angle into the gingival sulcus are essential in the probing examination, but the shape of the upper prosthesis in the implants has wide emergence profile which makes the appropriate probing difficult. In this study, there was no significant difference between probing depth and peri-implant inflammation in relation to implant marginal bone loss.

Walton et al. [34] and Payne et al. [35] reported that retention loss of attachment system was the most frequent complication. Similarly, replacement of the Locator male occurred most frequently in the IARPD group of this study. On the other hand, the ISBRPD group had the advantage that there was no decrease in retention. The retentive force by the clasp can be adjusted relatively easily by the dentist, and if there is no defect in the laboratory process, no adjustment is needed for a relatively long period. However, the maintenance frequency of the male of the Locator implant attachment is high due to wear that occurs during the attachment and detachment of the denture, as well as the functional load that occurs during mastication. Locator implant attachment is relatively easy to replace and maintain. Except for retention-related complications, there are no other specific complications in the two groups, and the incidence is less frequent than traditional removable partial dentures.

Due to the recent expansion of application range of removable partial dentures and implants in National Health Insurance, the implant-assisted partial denture is an increasing choice in treatment options for the partially edentulous patient. However, the implant-assisted removable partial dentures should be evaluated and selected based on the treatment plans of traditional partial dentures, and further considerations in selecting the number of implants, the location of the implants, and the shape of the connections are necessary. Also, the importance of each component of conventional partial denture should not be overlooked in the production of implant-assisted partial dentures, even though the implants assist the role of each component of conventional partial dentures.

Because of the limited number of subjects and short duration of the observation, this study does not show significant differences in the clinical indices between fixed prostheses used as abutments and in order to overcome these limitations and to provide a reliable clinical indices of the efficacy of fixed prostheses used as abutments, additional long-term studies from various institutions are needed.

## 5. Conclusions

In this study, implant marginal bone resorption was significantly higher in the IARPD group than in the ISBRPD group. However, ISBRPD group showed the higher frequency of calculus compared to the IARPD group. Overall clinical complications were higher in the IARPD group than in the ISBRPD group. Within the limits of the present study, it was found that well-planned ISBRPD was clinically appropriate. These results may be a pilot reference for implant-assisted removable partial denture with implant surveyed crowns, and longitudinal and systematic clinical studies are necessary to confirm these results.

## Figures and Tables

**Table 1:** Data of patients and implants.

Patient	Gender	Age (y)	Restored arch	Kennedy class	Implant connection type	Type of opposing dentition	Number of RPD abutments (implant)	Number of RPD abutments (natural teeth)	Follow-up period (month)
1	F	73	Mx	I	S	F	2	0	36
2	F	70	Mx	II	S	R	2	3	37
3	F	63	Mn	I	S	C	2	3	28
4	F	62	Mn	I	S	R	2	0	25
5	F	67	Mx	II	S	F	2	2	36
6	F	75	Mx	II	S	C	2	3	12
7	M	43	Mn	I	S	F	3	0	13
8	F	72	Mn	I	S	R	2	2	22
9	F	69	Mn	I	S	F	2	3	29
10	F	54	Mn	I	S	F	2	3	23
11	F	68	Mn	I	S	R	2	2	31
12	F	66	Mn	I	S	R	2	0	28
13	F	49	Mn	I	A	F	2	4	41
14	F	66	Mn	I	A	R	2	2	22
15	F	64	Mx	I	A	F	4	3	12
16	M	76	Mn	I	A	C	2	0	13
17	F	64	Mn	I	A	C	2	0	44
18	M	71	Mn	I	A	C	2	0	41
19	F	84	Mn	I	A	C	2	0	34
20	M	68	Mn	I	A	C	2	0	12
21	M	39	Mn	I	A	R	4	0	25
22	M	77	Mn	I	A	C	2	0	12
23	F	64	Mn	I	A	C	2	0	14
24	F	58	Mn	I	A	C	2	0	12

Implant connection type: S, surveyed bridges; A, attachment. Type of opposing dentition: R, removable partial denture; C, complete denture.

**Table 2:** Cumulative survival rate of the implants.

After placement (mo)	ISBRPD group			IARPD group		
	Implants (*N*)	Failed implants (*N*)	CSR (%)	Implants (*N*)	Failed implants (*N*)	CSR (%)
12~24	10	—	100	12	—	100
25~36	8	—	100	6	—	100
over 36	7	—	100	10	—	100

CSR: cumulative survival rate of implants.

**Table 3:** The average value of marginal bone resorption and probing depth.

	ISCRPD group		IARPD group		*p*
	Mean	SD	Mean	SD	
Marginal bone resorption (mm)	1.44	0.57	1.99	0.70	0.004^*∗*^
Probing depth (mm)	3.19	0.86	3.12	0.82	0.817

^*∗*^Mean values showed significant difference based on independent *t*-test (*p* < 0.05).

**Table 4:** Peri-implant inflammation, bleeding index, plaque index, and calculus.

Number of implants	ISCRPD group^†^	IARPD group^†^	*p*
	25	28	
Peri-implant inflammation (%)			
0	78.3	78.6	1.000
1	21.7	21.4	
2	—	—	
3	—	—	
Bleeding index (%)			
0	56.5	32.1	0.279
1	26.1	39.3	
2	17.4	28.6	
3	—	—	
Plaque index (%)			
0	47.8	28.6	0.121
1	21.7	50.0	
2	26.1	21.4	
3	4.4	—	
Calculus (%)			
0	69.6	96.4	0.016^*∗*^
1	30.4	3.6	

^†^Frequency distribution of gingival inflammation, bleeding index, plaque index, and calculus.

^*∗*^Frequency distribution showed significant difference based on chi-square test (*p* < 0.05).

**Table 5:** Type of clinical complication.

	ISBRPD group	IARPD group
Retention loss	0	14
Screw loosening	0	0
Resin base relining	4	3
Resin base repairing	2	5

Total	6	22
